# Soil Antibiotic Pollution and Ecological Risk Assessment in the Pearl River Delta Region, China

**DOI:** 10.3390/toxics13111004

**Published:** 2025-11-20

**Authors:** Yong’an Chen, Zhenxian He, Haochuan Wu, Xueqin Tao, Xiaolong Yu, Xiaojun Niu, Jianteng Sun

**Affiliations:** 1 School of Environmental Science and Engineering, Guangdong University of Petrochemical Technology, Maoming 525000, China; 2 Chemical Technology and Engineering Faculty, Belarusian State Technological University, 220006 Minsk, Belarus; 3 College of Resources and Environment, Zhongkai University of Agriculture and Engineering, Guangzhou 510225, China; 4 School of Environment and Energy, South China University of Technology, Guangzhou 510006, China

**Keywords:** antibiotics, Pearl River Delta, agriculture soil, spatial distribution, ecological risk

## Abstract

Excessive antibiotic use and their release into soils leads to alterations in soil processes, adversely affecting terrestrial organisms and presenting a serious threat to crop growth and productivity. Herein, the spatial patterns and prevalence of 22 typical antibiotics in agricultural soils throughout the Pearl River Delta area of China. In addition, the contamination characteristics and health risks were evaluated by integrating ArcGIS 10.7 spatial analysis, spearman correlation, and Risk Quotient (RQ) modeling. Antibiotics were detected in all the 240 soil samples with the total concentrations ranging from ND to 897 μg·kg^−1^ dry weight. The concentrations of seven antibiotic classes followed the order: quinolones (21.0 μg/kg) > β-lactams (15.1 μg/kg) > tetracyclines (9.65 μg/kg) > sulfonamides (3.91 μg/kg) > macrolides (0.26 μg/kg) > chloramphenicol (0.18 μg/kg) > lincosamides (0.03 μg/kg). Among the 240 sampling sites, quinolone antibiotics had the highest average contribution rate (41.89%), followed by β-lactams (30.09%), tetracyclines (19.28%), sulfonamides (7.81%), macrolides (0.52%), and chloramphenicol (0.36%), while lincomycin had the lowest contribution rate (0.06%). Spatial distribution demonstrated a significant positive correlation with per capita livestock/poultry product consumption, while 82.5% of sampling sites exhibited medium-high ecological risk primarily linked to livestock, poultry, and aquaculture. Quinolones, β-lactams, and tetracyclines were identified as the dominant ecological risk drivers in current regions. Mitigation requires synergistic measures including regulated veterinary drug use in aquaculture, enhanced manure treatment, and pollution remediation systems. These findings provide a scientific basis for supervising antibiotic pollution in agriculture soil and improving the sustainability and health of soil in the Pearl River Delta.

## 1. Introduction

Antibiotics have been instrumental in combating bacterial infections, lowering death rates, and increasing human longevity [1]. Nevertheless, the large-scale production and overuse of antibiotics have resulted in global antibiotic pollution and public health issues [2]. Globally, approximately half of antibiotics serve as veterinary drugs and feed additives, with 30–90% entering the various environment compartments via excreta due to their inadequate metabolism by animal and human guts [3,4]. In 2019, veterinary antibiotics usage reached 30,903.66 tons [5], predicting that China will remain the largest global consumer by 2030 [6]. Antibiotics have been frequently detected in aquatic environments. For instance, ciprofloxacin, amoxicillin, and azithromycin were detected in the Kabul River (Pakistan), the Mijares River (Spain), the Oujiang River Basin (Zhejiang, China), and the Chaohu Lake Basin (Anhui, China) [7,8,9,10], and were widespread in groundwater. Han et al. identified 23 antibiotics in Hutuo River alluvial fan ground water (North China Plain), with total concentrations ranging from ND to 465.26 ng/kg [11]. Quinolones predominated in all detected samples (mean:12.66 ng/L), and the major quinolones were ciprofloxacin and ofloxacin. Li et al. reported the high detection frequency of antibiotics (42.9–80.4%) in Han River groundwater, where gentamicin peaked at 9.3 ng/L [12].

Soil as a critical reservoir accumulates various antibiotics through fertilization, wastewater irrigation, sludge reuse, and other pathways, with escalating pollution concerns [13]. Antibiotics primarily enter soils via manure application, sewage irrigation, and sludge disposal [14]. Bao [15] reported that total antibiotic concentrations ranged from ND to 609.62 μg/kg in eastern Inner Mongolia agricultural-pastoral soils, and tetracyclines accounted for the dominant abundance. Leal [16] detected the high level of quinolones (22.93 μg/kg) in São Paulo soils. Residual antibiotics could disrupt microbial communities function [17], plant metabolism [18], and the growth and development of soil animals [19]. Antibiotics are also prevalent in feces, vegetables, aquatic products, and sediments [20,21,22,23], and secondary contamination risks of antibiotics towards agricultural soils could be caused through fertilization.

Antibiotic residues in soil may promote the emergence of novel antibiotic-resistant bacteria (ARB) and resistance genes (ARGs), thereby impeding pathogen control and posing public health risks [24,25]. Previous studies have shown that antibiotics can enter organisms through food and water, disrupting gut microbiota balance and causing intestinal inflammation [26,27]. Additionally, soil microbial communities structure and composition were destroyed by antibiotics, facilitating horizontal gene transfer of resistance genes, and endangering ecosystems and public health [28,29,30]. Their adverse effects involve inhibiting the transcription and translation [31], protein synthesis and modification [32], and disrupting cell membrane permeability and cellular energy metabolism [33,34]. Consequently, a comprehensive assessment of current antibiotic contamination levels and their spatial distribution patterns in soil of the Pearl River Delta (PRD) is essential for the accurate ecological and public health risk evaluation of them.

Risk assessment of emerging contaminants as an effective prediction approach could afford the scientific basis for their supervision and management, which encompasses health and ecological evaluation. Health risks are commonly evaluated via hazard quotient (HQ) and hazard index (HI) for ingestion pathways, while ecological risks predominantly employ the risk quotient (RQ) method [35]. RQ thresholds are: <0.1 (low risk), 0.1–1 (medium risk), and >1 (high risk) [36]. Although vegetable-derived antibiotic health risks appear minimal [37], ecological risks in soils remain significant across multiple regions. The PRD located in the south-central Guangdong Province along the South China Sea, is one of the world’s most populous and economically vibrant regions. Its strong advanced manufacturing and labor-intensive agriculture sectors may increase the region’s environmental pressures. Antibiotic residues in various vegetables in PRD have been documented repeatedly [38,39]. Pan et al. observed that the antibiotic concentrations in fish pond water-irrigated soils were higher than sewage-irrigated soils across the PRD [40]. Elevated detection rates of antibiotics in Pearl Estuary rivers verified substantial aquatic ecological risk and potential public health risk [41]_._

The objectives of this research were to: (i) determine the levels and compositional profiles of antibiotics in PRD agricultural soils; (ii) analyze the spatial distribution patterns and dominant antibiotic sources; (iii) quantify carcinogenic and non-carcinogenic health risks posed by antibiotic exposure to residents (adults and children) via dietary intake and environmental pathways. The findings will provide essential data for antibiotics contamination and crops safety in PRD, enhancing the scientific management of these antibiotics.

## 2. Materials and Methods

### 2.1. Chemicals and Reagents

A total of seven antibiotic standards were employed for chemical analysis. Sulfonamide antibiotics comprised sulfadiazine, sulfadimethoxine, sulfamethoxazole, sulfamerazine, and sulfamethazine, with standards (purity ≥ 98%) obtained from Shanghai Yuanye Biotechnology Co., Ltd (Shanghai, China). Quinolone antibiotics included ofloxacin, enrofloxacin, norfloxacin, lomefloxacin, and flurofloxacin (purity ≥ 98%, Shanghai Yuanye Biotechnology Co., Ltd.), alongside moxifloxacin (purity ≥ 98%, Shanghai Aladdin Biochemical Technology Co., Ltd (Shanghai, China). β-lactam antibiotics consisted of amoxicillin, cephalexin, and cefotaxime (purity ≥ 97%, Shanghai Yuanye Biotechnology Co., Ltd.) and cefaclor (purity ≥ 97%, Shanghai Aladdin Biochemical Technology Co., Ltd.). HPLC-grade formic acid, methanol, and acetonitrile were sourced from Shanghai Aladdin Biochemical Technology Co., Ltd. Milli-Q ultrapure water was used throughout the experiments, and other chemical reagents were presented in Supporting Information Text S1.

### 2.2. Sample Collection and Preparation

**Figure 1:**
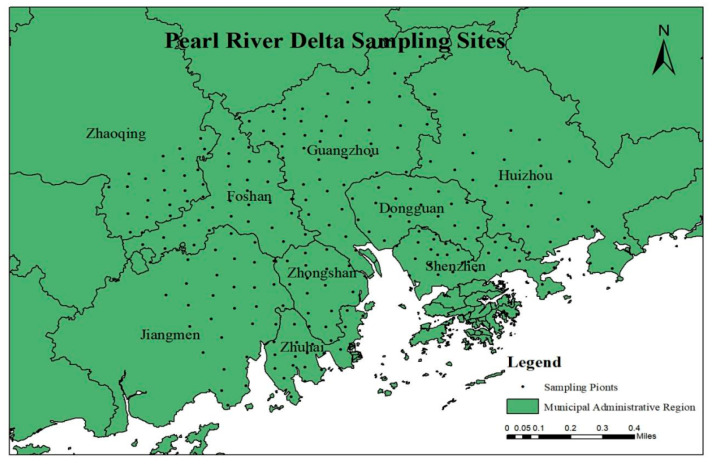
Locations of study area and field sampling sites in the Pearl River Delta region.

A total of 240 soil samples were collected from vegetable fields across nine cities in the Pearl River Delta region, including Guangzhou (48), Shenzhen (30), Dongguan (18), Jiangmen (30), Zhongshan (16), Huizhou (28), Zhaoqing (24), Zhuhai (19), and Foshan (27), as shown in Figure 1. To ensure sample representativeness, an S-shaped distribution pattern was employed at each sampling site. Five surface soil subsamples (0–15 cm depth) were uniformly collected using stainless steel shovels and then they were merged into a single sample (~1 kg) [42]. Samples were immediately stored in aluminum foil-lined kraft paper bags under refrigeration, and GPS coordinates were recorded for all sampling sites. Soil samples pretreatment involved three steps: natural air-drying, impurity removal, and manual grinding followed by sieving. The prepared soil samples were analyzed for pH and total organic carbon (TOC) following the potentiometric method [43] and the potassium dichromate volumetric method [44], respectively. Briefly, the soil pH was determined using a pH meter (Inesa, Shanghai, China) at a soil-to-water ratio of 1:2.5. As to the TOC, soil samples need to undergo pretreatment steps such as air drying, grinding, and sieving before detection to ensure the uniformity and representativeness of the samples. For soils containing carbonates, inorganic carbon should be removed by acidification or high-temperature burning to avoid interference with TOC determination. Afterwards, the TOC was analyzed using an Elementar Vario EL III elemental analyzer (Hanau, Germany).

### 2.3. Antibiotics Extraction

A total of 1 g of soil was weighed into a 10 mL centrifuge tube. After introducing 0.2 g EDTA and 5 mL extraction solution (prepared with 27.2 mL KH_2_PO_4_ and 1.35 mL H_3_PO_4_ fixed to 1 L with ultrapure water, then mixed 1:1 with acetonitrile, pH 3.0), samples were vortexed (1 min) and underwent ultrasonication for 20 min, and then centrifuged (2500 r/min, 5 min). The supernatant was harvested and the extraction was repeated three times. Combined supernatants were diluted to 300 mL with ultrapure water and passed through Oasis HLB columns (activated with 6 mL methanol and 6 mL ultrapure water) at a 5 mL/min flow rate. Columns were rinsed with 15 mL ultrapure water and vacuum-dried (10 min). The obtained samples were diluted with 9 mL methanol containing 0.1% formic acid into centrifuge tubes to evaporate and then be reconstituted with methanol to 1 mL. Finally, solutions were filtered through 0.22 μm syringe filters into amber vials for quantitative analysis. The detailed experimental procedure can refer to the relevant method [45].

### 2.4. Instrumental Analysis

Antibiotics quantification analysis was performed by UPLC-MS/MS system (Thermo Scientific Dionex UltiMate 3000 Series) (Thermo Fisher Scientific, Sunnyvale, CA, USA) with a ACQUITY BEH C18 column (2.1 mm × 50 mm × 1.7 μm, Phenomenex, Torrance, CA, USA) in positive mode via multiple reaction monitoring (MRM). The mobile phases consisted of (A) 0.1% formic acid in water and (B) methanol–acetonitrile (2:8, *v*/*v*) containing 0.1% formic acid with a total flow rate of 0.2 mL/min. A volume of 10 μL was injected and column temperature was 30 °C. The gradient program was as below: 0–2 min (95% A), 2–4 min (85% A), 4–6 min (75% A), and 6–7 min (5% A). An electrospray ionization (ESI) source and selected ion monitoring (SIM) mode with a resolution of 70,000 were selected for chemicals detection. The capillary temperature was set as 320 °C. Auxiliary gas heating temperature was set as 350 °C and sheath gas pressure was 275.8 kPa. Auxiliary gas pressure and spray voltage was set as 68.95 kPa and 3.0 kV, respectively. The limits of detection (LODs), calculated as signal-to-noise ratios of 10, were 0.03 to 0.06 μg/kg for antibiotics. Antibiotic quantification was performed using external calibration curves prepared in methanol. Method reliability was validated through quality control analysis, which involved incorporating spiked samples with known concentrations into each batch. The relevant analytical parameters of MS for antibiotics detection and the calibration details were presented in Supporting Information (Table S1 and Text S2).

### 2.5. Quality Control

Quality control measures included processing method blanks, spiked samples, and analytical replicates at 10-sample intervals to ensure contamination-free procedures and analytical accuracy. Antibiotics were not detected in all blanks, with experimental samples concentrations showing <10% relative standard deviation (RSD). Target chemicals recoveries (spiked at 1, 5, 10, and 20 μg/kg) were maintained in a range of 82–116%. Calibration curves exhibited R^2^ > 0.99.

### 2.6. Ecological Risk Assessment

The ecological risk assessment follows the Risk Quotient (RQ) value method in the EU Technical Guidance Document (TGD) on Risk Assessment of the European Commission (2003) [35]. RQ < 0.1 is considered as low risk, 0.1 ≤ RQ ≤ 1 is considered as medium risk, and RQ > 1 is considered as high risk [36]. The selection of AF in this study was set to 1000, which referred to the EU Technical Guidance Document on chemical risk assessment issued by the European Chemicals Agency [35]. The RQ is calculated using the following formula:

RQ = MEC/PNEC_water/soil_ (1)

where MEC is the measured environmental concentration of antibiotics; PNEC_water_ is the predicted no-effect concentration of antibiotics in water. According to the EU Technical Guidance Document [35] and the calculation method described by Ren [46], the PNEC_soil_ for antibiotics in soil was obtained by equilibrium conversion using the PNEC_water_ for antibiotics in existing studies with the following equation:

PNEC_soil_ = K_soil–water_ × PNEC_water_ (2)

K_soil–water_ = F_water–soil_ + F_solid–soil_ × (KP_soil_ × RHO_solid_/1000) (3)

logK_oc_ = 0.623logK_ow_ + 0.873 (4)

where K_soil–water_ is the organic matter soil-water partition coefficient; F_water–soil_ and F_solid–soil_ are the volume fractions of the water and solid phases of the soil, respectively (standard environmentally defined values in the TGD were used in this study, which were 0.2 and 0.6, respectively); RHO_solid_ is the solid density (value of 2500 kg·m^−3^ defined in TGD); KP_soil_ is the solid-phase water partition coefficient, which was calculated using 0.02 times the organic matter–soil–water standardized carbon (*Koc*) based on the TGD recommended values; *Kow* is the octanol-water partition coefficient. The mixture risk quotient (MRQ) due to co-contamination of different antibiotics was assessed using the following model:

MRQ = Σ(MEC_i_/PNEC_i_) (5)

As the PNEC_water_ values for some substances are less frequently reported, only 16 antibiotics were risk assessed in this study, with PNECs derived from Ren [46].

### 2.7. Spatial Mapping and Statistical Analysis

Statistical analyses were performed using Origin 2021 (OriginLab Inc., Northampton, MA, USA) and SPSS 21.0 (IBM, Armonk, NY, USA). All data is presented as mean ± standard deviation (SD). In addition, Origin 2021 was also used to analyze data on concentrations of contaminants and risk quotients and to plot correlation analyses. ArcGIS 10.7 software was employed to map and analyze the spatial distribution of antibiotics in soil within the sampling area. Relationships between antibiotic concentrations in soil, TOC in soil, and pH will be assessed using Spearman’s correlation analyses.

## 3. Results and Discussion

### 3.1. Characteristics of Antibiotics in Soil in the PRD

Based on the analysis of 240 PRD vegetable field soil samples, (Table 1) total antibiotic levels ranged from ND to 897 μg/kg, with a mean value of 50.0 μg/kg. A total of 22 antibiotics were determined, including sulfonamides, quinolones, tetracyclines, beta lactams, macrolides, lincomycin, and chloramphenicol. The total concentration of these antibiotics was <LOD~328 μg·kg^−1^, <LOD~870 μg·kg^−1^, <LOD~106 μg·kg^−1^, <LOD~99.1 μg·kg^−1^, <LOD~6.52 μg·kg^−1^, <LOD~0.33 μg·kg^−1^, and <LOD~6.50 μg·kg^−1^, with detection rates being 86.3%, 70.0%, 89.2%, 70.8%, 64.6%, 30.0%, and 3.8%, respectively (Table S2). Mean concentrations of these antibiotics followed the order: quinolones (21.0 μg/kg) > β-lactams (15.1 μg/kg) > tetracyclines (9.65 μg/kg) > sulfonamides (3.91 μg/kg) > macrolides (0.26 μg/kg) > chloramphenicol (0.18 μg/kg) > lincosamides (0.03 μg/kg). Compositional contributions to total antibiotics were as follows: quinolones (41.89%), β-lactams (30.09%), tetracyclines (19.28%), sulfonamides (7.81%), macrolides (0.52%), chloramphenicol (0.36%), and lincosamides (0.06%).

Sulfonamide antibiotics exhibited high detection frequencies (10.0–73.8%) in PRD soils. Sulfadimethoxine was identified as the primary contributor (2.93 μg/kg), followed by sulfadiazine, sulfamethoxazole, sulphadimethoxine, and sulfamerazine with average concentrations of 0.39 μg/kg, 0.24 μg/kg, 0.23 μg/kg, and 0.12 μg/kg. Notably, the mean concentration of sulfonamide antibiotics in PRD soils (3.91 μg/kg) significantly exceeded the levels of them in the Yangtze River Delta (2.35 μg/kg) [42] and Zhangjiagang farmland (2.08 μg/kg) [46], potentially attributing to intensive veterinary antibiotic use in PRD agriculture. Detection frequencies of quinolones ranged from 2.5 to 58.3%, with norfloxacin emerging as the dominant risk chemical (mean: 10.1 μg/kg, maximum: 684 μg/kg). The mean concentration of quinolone (21.0 μg/kg) surpassed the levels of them in Guangzhou organic farms (14.0 μg/kg) [47] and Nanjing greenhouse soils (<LOD–649 μg/kg) [48]. Critically, the peak concentration of norfloxacin approached seven-fold the ecotoxicity threshold (100 μg/kg), enhancing our vigilance regarding long-term cumulative effects. Tetracyclines demonstrated 40.0–63.8% detection frequencies, dominated by oxytetracycline (4.15 μg/kg). Their overall concentrations were lower than those in Guangzhou’s green/organic vegetable bases (0.98–6.59 μg/kg) [49] and Beijing’s greenhouse vegetable production bases (102 μg/kg) [50], likely reflecting tightened tetracycline usage controls in the PRD. Amoxicillin (8.52 μg/kg) and cefadroxil (5.84 μg/kg) that were classified in β-lactam antibiotics in PRD exhibited a notable high concentration, signifying the potential contamination from urban medical waste or human antibiotic residues. Macrolide antibiotic concentrations were generally low in this work. However, peak values of azithromycin (6.52 μg/kg) and methicillin (6.50 μg/kg) exceeded Chongqing soil levels by 2–4 times, elucidating that the continuous monitoring of sites with anomalies should be proceeded [51]. According to the International Coordinating Committee for Veterinary Medicine, antibiotics concentrations in soil exceeding the safety threshold (100 μg/kg) may induce ecotoxicological effects on various organisms [41]. In current work, 10.4% of samples exceeded the threshold for total antibiotics, while 5.8% exceeded for single antibiotic classes and 4.6% for individual compounds. Norfloxacin, enrofloxacin, and sulfamethazine constituted the primary contaminants in samples that exceeded the ecotoxicity threshold, reflecting regional veterinary antibiotic usage patterns in the PRD. Furthermore, these contamination hotspots pose potential threats to soil microbial communities and may facilitate resistance gene dissemination. The previous study conducted a quantitative analysis of 13 antibiotic residues in 241 soil samples collected from the Yangtze River Delta region. The results showed that the concentration range of total antibiotic was 4.55–2010 ng/g (dry weight), with an average concentration of 86.1 ng/g [42]. Quinolones exhibited a substantially higher average concentration (48.8 ng/g) compared to tetracyclines (34.9 ng/g) and sulfonamides (2.35 ng/g), which was consistent with our findings. Quinolones, tetracyclines, and sulfonamides were identified as the predominant antibiotics in agricultural soils, where their corresponding resistance genes were also prevalent [52,53,54]. The concentrations of quinolone antibiotics in this study (ND-458.3 ng/g) were similar to the levels of them in arable greenhouse soils in Nanjing (below LOD-290 ng/g) and Beijing (below LOD-649 ng/g) [48,50], but were substantially lower than those observed in the vegetable farm soils of the Pearl River Delta (27.8–1530 ng/g) [39], elucidating the regional variation in antibiotic pollution. The average concentration of sulfonamide antibiotics detected in this work (3.91 ng/g) was significantly higher than the reported level in the Huanghuaihai Plain (<0.11 ng/g) [55]. Wei [56] found that average antibiotic residue levels in Hebei, Henan, Sichuan, and Jiangsu agricultural soils followed the order: tetracyclines > quinolones > macrolides > sulfonamides > benzophenols. Although antibiotics generally occur in soil at lower concentrations than typical soil pollutants, fluoroquinolones and tetracyclines have been detected at the range of mg kg^−1^ [57]. The level of quinolone antibiotics in farm soil in Cordoba, Spain reached 15.6 mg/kg [58].

Rapid urbanization and intensive agricultural practices driven by urban expansion have significantly impacted residue levels of antibiotics in soil. Antibiotics contamination has been increasingly found in greenhouse vegetable soils and mulched soils, making them priority areas of concern. On the one hand, the extensive use in the breeding industry (livestock breeding and aquaculture) of veterinary medicines in this area and the overuse of antibiotics in clinic settings resulted in higher abundance of antibiotics. On the other hand, sediments, sewage sludge, and biosoilds containing antibiotics because of the limited elimination efficiency of wastewater treatment plants, are also commonly used as fertilizers in agricultural activities, and are applied to agricultural fields in South China [59,60,61], eventually leading to excessive residuals of antibiotics in vegetable soils in the PRD region.

### 3.2. Characteristics of Spatial Distribution of Antibiotics in the PRD

**Figure 2:**
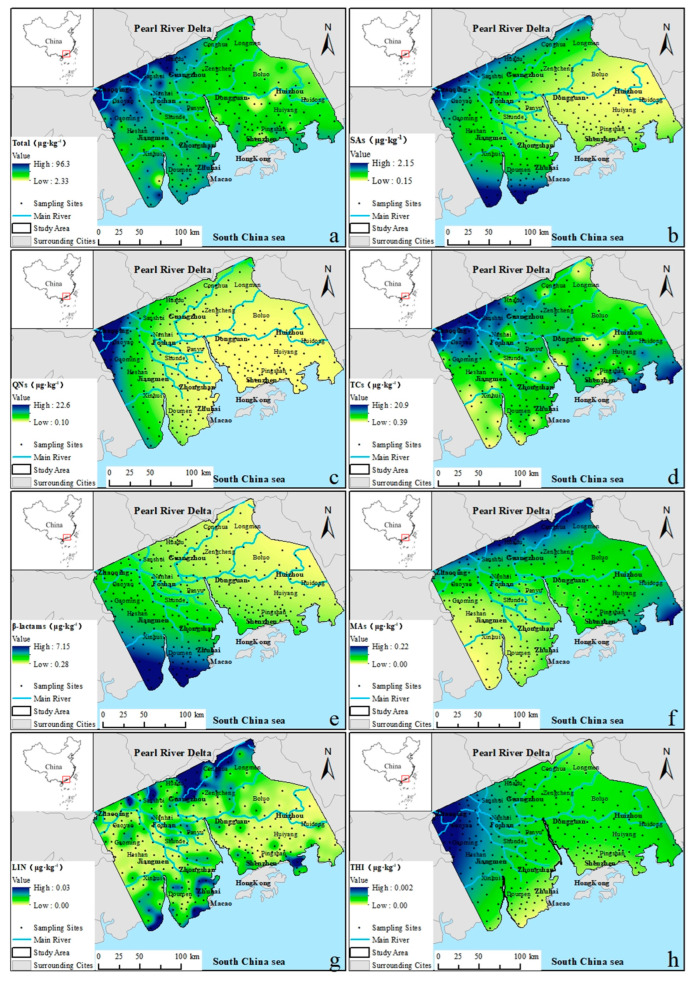
Spatial distributions of (**a**) total antibiotics; (**b**) sulfonamides; (**c**) quinolones; (**d**) tetracyclines; (**e**) β-lactams; (**f**) macrolides; (**g**) lincomycins; (**h**) thiamphenicols in farmland soils of the Pearl River Delta region.

The pollution characteristics of antibiotics exhibit regional variations, as their sources are closely associated with urban activities and agricultural practices involving sewage sludge and biosoilds. To visualize the concentration distribution, the spatial distribution of soil antibiotics was estimated using the Simple Kriging interpolation method within ArcGIS 10.7’s spatial interpolation module, with data processing confined to the sampling area. As illustrated in Figure 2, the total antibiotics concentrations in farmland soils of the Pearl River Delta exhibited high levels in the following areas: Zhaoqing, northern and western Foshan, northern Guangzhou, southern Jiangmen, as well as southern and eastern Zhuhai. This was attributed to the fact that many companies, such as electrical appliances, furniture, textiles, were concentrated in above regions, which increased the usage amount of antibiotics by native residents and migrant workers due to the labor population. Finally, high levels of antibiotics in domestic sewage contribute to the accumulation in sewage sludge and effluents of wastewater treatment plants, which aggravates the emission of antibiotics into agricultural fields during their cycling process. In addition, accelerated urbanization, in conjunction with agricultural and domestic waste, has been associated with increased antibiotic concentrations in this region [62]. For instance, the rising demand of antibiotics by residents buying through retail pharmacies and private clinics could result in the overuse and misuse of antibiotics, which cause unreasonable disposal and increase the leakage of antibiotics into the environment. Moreover, developed livestock breeding and aquaculture in the above regions are also deemed as pivotal sources for the emission of antibiotics because of the poor removal efficiency of the high concentration of antibiotics in wastewater. Moderate level values occurred in central Foshan, northern Jiangmen, central Zhongshan, western/southeastern Shenzhen, and southern Huizhou. In addition to the above areas, antibiotic concentrations from other sample sites displayed a low concentration distribution. Overall spatial patterns manifested: (1) higher concentrations in central-western compared to eastern regions; (2) greater concentrations in northern areas compared to southern areas (Figure 2a). Total concentrations in Zhaoqing, Foshan, Jiangmen, Zhongshan, and Zhuhai exceeded those in Guangzhou, Dongguan, Shenzhen, and Huizhou (*p* < 0.05), in accordance with the finding of Yang [63].

Among the specific classes, sulfonamides accumulated primarily in northern, northwestern, and southwestern regions (Figure 2b), with high-concentration areas significantly correlating with districts reporting elevated agricultural (*p* < 0.05), pastoral, and fishery output values in statistical yearbooks of Guangzhou (2024), China. Quinolones and thiamphenicols concentrated in northwestern sectors, yet the estimated maximum concentration of thiamphenicols was 0.002 μg·kg^−1^ (Figure 2c,h). Tetracyclines with high concentration was discovered in the northwest/southeast area of the current research zones (Figure 2d). β-lactams predominantly accumulated in southwestern areas (Figure 2e). Macrolides and high levels of lincosamides showed in the northeast/southeast regions (Figure 2f), and the high value area of lincomycin was located in northern/southern zones (Figure 2g). Yearbook data of Guangzhou (2024) confirmed that quinolone, tetracycline, β-lactam, and macrolide hotspots frequently coincided with high-output regions including Taishan, Gaoyao District in Zhaoqing, and Huizhou.

### 3.3. Potential Sources Apportionment of Antibiotic Contamination in the PRD

Soil antibiotic contamination in the Pearl River Delta region is shaped by multiple factors, including pollution discharge sources and the physicochemical properties of the antibiotics themselves, e.g., their adsorption characteristics and environmental persistence. Apart from wastewater irrigation, agricultural practices like fertilization and pesticide application also play significant roles. For instance, long-term application of organic fertilizers, including biogas slurry and manure, has been identified as a key driver of antibiotic accumulation in agricultural soils. Zhang [64] reported widespread distribution and elevated levels of veterinary antibiotics in manured soils, and the high usage amount of organic fertilizer application will aggravate the accumulation of veterinary antibiotics in soil [65]. Shi [66] identified a dose–response relationship between manure application and tetracycline accumulation in soils, where high application rates (80.44 t/ha) yielded concentrations 4.1 times those of low-rate soils (20.11 t/ha). This phenomenon is consistent with broader observations that long-term application of organic fertilizers, including biogas slurry and manure, leads to elevated antibiotic levels in agricultural soils, with accumulation patterns showing spatial and temporal variability [67]. Sulfonamide antibiotics serve as key elements in veterinary medications and feed additives. The large scale of livestock breeding and aquaculture in the Pearl River Delta region has surged the usage of sulfonamide antibiotics, which has increased the accumulation of them in municipal wastewater and sewage sludge. Moreover, sulfonamide antibiotics are the most commonly found antibiotics in farm wastewater and manure [68]. Some sampling sites located in family orchards in rural areas of Zhaoqing, Foshan, Guangzhou, Jiangmen, and Zhuhai exhibited high detection rates of sulfonamide, quinolones, and tetracyclines, which might be due to the composting of manure by rural retail farming properties. In addition, the high abundance of antibiotics in Jiangmen, Zhuhai, Zhongshan, and Huizhou might be ascribed to manure application in the field, or irrigation with aquaculture wastewater. As reported by a previous study, municipal sewage is the main source of antibiotics in the soil around urban areas, especially quinolone antibiotics [69]. In addition, a survey on soil antibiotic pollution around the Beijing, Tianjin, Hebei, Jiangsu, Zhejiang, and Shanghai urban agglomerations in China showed that sewage irrigation and organic fertilizer application are the main sources of soil antibiotic pollution in these areas [70]. Overall, wastewater irrigation, manure application of sewage sludge, and organic fertilizers (biogas slurry and manure) are considered to be main contributors to the high levels of antibiotic pollution in the PRD region.

### 3.4. Correlation of Contaminant with Soil Properties and Livelihood

The Pearl River Delta is a highly developed agricultural production area and population concentration area in Guangdong Province. According to statistical yearbooks of Guangzhou (2019), China, its pig slaughter volume, poultry slaughter volume, aquatic products production, Gross Agricultural Output Value, and permanent resident population accounted for 26.8%, 24.9%, 39.5%, 31.1%, and 50.9% of the provincial totals, respectively. Correlation analysis between these items and the mean total antibiotic concentration in soil (including the mean concentrations of seven specific antibiotic classes, such as sulfonamides and quinolones) revealed significant associations. As depicted in Table 2, the mean total soil antibiotic concentration showed significant positive correlations with pig slaughter volume (r = 0.833, *p* < 0.01), poultry slaughter volume (r = 0.717, *p* < 0.05), and aquatic products production (r = 0.917, *p* < 0.01), indicating that livestock farming and aquaculture were primary pollution sources. Correlations with Gross Agricultural Output Value (r = 0.617, *p* = 0.077) and permanent resident population (r = −0.400, *p* = 0.286) were not statistically significant. Quinolones correlated significantly with aquatic products production (r = 0.733, *p* < 0.05), suggesting that the excessive usage of aquaculture drugs might be a source. Thiamphenicol correlated significantly with Gross Agricultural Output Value (r = 0.797, *p* < 0.05), indicating the contributions of agricultural drug source. β-Lactams exhibited a significant negative correlation with permanent resident population (r = −0.850, *p* < 0.01), suggesting that human antibiotics were unlikely their main source. We deduced that the overuse of β-Lactams in livestock farming might contribute to its high abundance. Furthermore, the mean concentrations of sulfonamides, tetracyclines, macrolides, and lincomycin showed no significant correlations with any of the five socio-economic indicators, implying a low probability that their contamination stems directly from the current use of agricultural, livestock, aquaculture, and human antibiotic.

**Figure 3:**
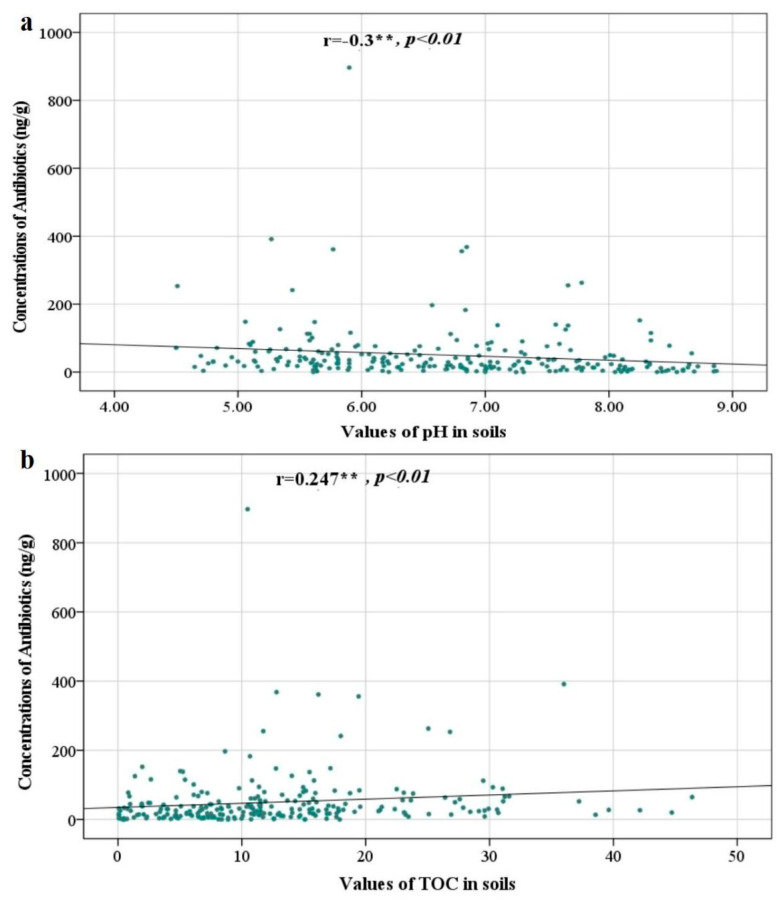
Correlation between total antibiotics and pH (**a**) and TOC (**b**) in farmland soils of the Pearl River Delta.

The distribution characteristics and migration patterns of pollutants in soil are not only related to their own properties but may also be correlated with the physicochemical properties of soil (e.g., pH, temperature, humidity, porosity and soil organic matter, etc.). Therefore, it is necessary to evaluate the physicochemical properties of soil samples and analyze the correlation between antibiotics levels and these environmental factors. In this study, the correlation between pH and soil TOC content of soil samples and antibiotic distribution characteristics in the PRD was analyzed and determined. The pH values of 240 samples remained in the range of 4.5–8.87, while the TOC values of these samples remained in the range of 0.08–46.36. As displayed in Figure 3a, a significant negative correlation was observed between the total antibiotic concentration and soil pH (r = −0.3, *p* < 0.01), indicating that the accumulation of antibiotics tends to decrease with increasing soil pH. This suggests that antibiotics may persist more readily in relatively acidic soil conditions. The underlying reasons may involve the influence of pH on the adsorption behavior, degradation rates, and microbial activity related to antibiotics. A higher pH environment might promote the hydrolysis or microbial degradation of certain antibiotics, thereby reducing their residual levels in the soil. Conversely, a significant positive correlation was found between the total antibiotic concentration and total organic carbon (TOC) (r = 0.247, *p* < 0.01), as illustrated in Figure 3b. This implies that soil organic matter content likely facilitates the accumulation of antibiotics. The mechanism may be that TOC enhanced the soil’s adsorption capacity for antibiotics through various mechanisms, such as hydrophobic interactions, which could subsequently retard their degradation or migration, leading to greater enrichment of antibiotics in soils with higher organic matter content.

Antibiotic accumulation in soils can be influenced by various soil properties, such as pH, soil organic matter (SOM), and soil component, therein affecting the microbial activities, adsorption, and metabolism process. Soil pH influences the environmental behavior of antibiotics by modulating their interactions with soil particles. The rising soil pH leads to a significant decrease in the concentrations of quinolones, macrolides, and tetracyclines in the soil. This occurs because higher pH reduces the acid dissociation constant (*pKa*) of these antibiotics, weakening their electrostatic attraction to soil particles and thereby diminishing their adsorption onto soil colloids [71]. For instance, including TCH^3+^ (pH < 3.3), TCH^−^ (7.7 < pH < 9.7), and TC^2−^ (pH ˃ 9.7) [72]. Tetracycline, as an amphoteric compound with three ionizable groups exhibiting distinct pKa values, demonstrates pH-dependent solubility behavior. Specifically, at higher pH levels, the anionic antibiotics exert more solubility in soil solution, reducing their adsorption affinity for soil particles [73]. In addition, the elevated pH may enhance the hydrolysis and biotransformation of antibiotics through stimulating microbial uptake and metabolism in the soil [74]. Consequently, the high soil pH levels in some sampling sites in the Pearl River Delta progressively diminish the adsorption capacity of antibiotics while simultaneously accelerating their degradation, ultimately resulting in a reduced number of antibiotics binding to soil sorption sites. A similar finding of antibiotics abundance being affected by pH values was reported by previous research [75]. Due to the abundant supply of carbon and energy sources for these microorganisms in cases of high TOC in soil, enhanced abundance and activities of microorganisms could promote high-efficient biodegradation of antibiotics, thereby reducing the accumulation of antibiotics in soil [76]. Moreover, iron-aluminum oxides can coordinate with quinolones to form five-membered ring chelates via surface complexation, while hydrophobic antibiotics like macrolides tend to adsorb onto the non-polar sites of soil organic matter. As a result, the distinction in soil physicochemical properties is another major driving factor which leads to differences in antibiotic distribution.

### 3.5. Health Risk Assessment

Health risk assessment of antibiotics is mainly employed to predict the risk that antibiotics in the environment may pose to humans [50]. The current study mainly focuses on assessing the potential health threats that may arise from human exposure to or ingestion of water, plants, and meat containing antibiotics through the hazard quotient (HQ) and hazard index (HI). HQ or HI < 0.01 is considered ‘no risk’. There is a significant risk when HQ ≤ 0.01 or HI < 0.05, and a serious risk when HQ or HI ≥ 0.05. ‘low risk’ when 0.01 ≤ the proposed value of the risk quotient is less than 0.1, and between 0.1 and 1 is medium risk [77].

**Figure 4:**
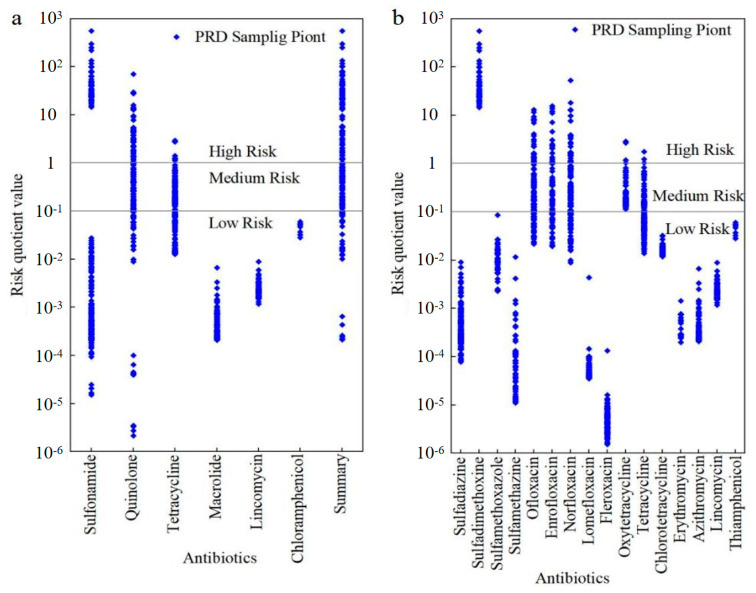
The risk quotient values of (**a**) six kinds of antibiotics and total antibiotics and (**b**) 16 kinds of antibiotics in soil of the Pearl River Delta region.

The MRQ of total antibiotics in agricultural soils in the PRD region ranged from 0.00 to 563.75, with a mean value of 16.50 (Figure 4a). The results elucidated that the percentages of high, medium, and low risk sites accounted for 45%, 37.5%, and 17.5%, respectively. Sulfonamide antibiotics had the largest average MRQ (14.60), resulting in 25.0% of the sampling sites with high risk. The ecological risk of sulfonamide antibiotics in agricultural soils in the PRD region was mainly caused by sulfadimethoxine. The maximum risk value for sulfadimethoxine was 563.74 and the mean value was 14.6 (Figure 4b). Ofloxacin, enrofloxacin, norfloxacin, oxytetracycline, and tetracycline also made certain sites with high MRQs, and approximately 21.7% of the high-risk sites were caused by their contamination. Other antibiotics exhibited low risk or no risk. Quinolones, tetracyclines, and sulfonamides have the main contributions to MRQ, with average contribution rates of 39.2%, 31.4%, and 25.4%, respectively. Six antibiotics, including sulfamethoxazole, ofloxacin, enrofloxacin, norfloxacin, oxytetracycline, and tetracycline, are the main antibiotics causing MRQ > 1, with a contribution rate of 41.7%. The average contribution of their pollution concentration in high-risk areas is 49.3%. The previous research confirmed that tetracyclines and quinolone antibiotics were the main contributors to soil antibiotic contamination in agricultural soils in the Yangtze River Delta region [42]. Tetracyclines, quinolones, and sulfonamides were the main antibiotics that constituted a medium-to-high risk in the soils of vegetable fields in Chongqing Municipality [51], which was consistent with the current results. Of note, the above studies emphasized that tetracyclines and quinolones were deemed as pivotal sources of antibiotics in agricultural soils. We deduced that this common result may stem from the dense population, labor-intensive agricultural activities, and the developed manufacturing industries of these areas. As such, the continuous monitoring for antibiotics in agricultural soil in these areas should be intensified for ensuring crops and vegetables safety.

## 4. Conclusions

This work revealed the antibiotic residues and health risk in agricultural soils in the Pearl River Delta region. Higher concentrations of antibiotics were detected in areas with more intensive agricultural and livestock activities, e.g., Zhaoqing, Foshan, Zhuhai, Guangzhou, and Jiangmen. This unmasked that the rapid developing livestock industry and urbanization in the Pearl River Delta region may cause the overuse and emissions of antibiotics. Total antibiotic concentrations in the Pearl River Delta region were negatively correlated with soil pH, and most soils were positively correlated with TOC. The ecological and health risk assessment elucidated that 82.5% of the sampling sites were medium/high-risk sites, implying dietary exposure risk and crops food safety. As a result, the antibiotic pollution in agricultural soils in the Pearl River Delta region should focus on, including the strict supervision of antibiotic use, the cycle of sewage sludge, and safe disposal of pharmaceutical wastewater. Nevertheless, the bioaccumulation and metabolic behavior of antibiotics in vegetables was not concentrated in this work, which will enlarge the understanding of the potential hazards to human health and ecosystem security of antibiotics. Thus, we proposed selecting crop varieties that accumulate fewer antibiotics and adopting biofertilizers with antibiotic-degrading bacteria to decrease antibiotic buildup in agricultural products. Additionally, microplastics can serve as carriers due to their physical and chemical properties, which can adsorb organic pollutants such as antibiotics and organophosphate esters to form combined pollution. This will promote the migration and diffusion of pollutants in the environment through the “Trojan Horse effect”, exacerbating the threat to ecosystems and human health. The presence of microplastics provides unique habitats for microorganisms and may become a “hotspot” for the spread of antibiotic resistance genes (ARGs). In future work, the combined toxic effects and environmental behavior of microplastics and antibiotics in soil–plant systems will be explored because of the limited relevant data in agricultural soils in the Pearl River Delta region.

## Figures and Tables

**Table 1:** Concentration and detection rate of antibiotics in vegetable soil of PRD region, China.

Chemicals	Max (μg·kg^−1^)	Min (μg·kg^−1^)	Median (μg·kg^−1^)	Mean (μg·kg^−1^)	Detection Rate (%)
Sulfadiazine	6.75	ND ^a^	0.20	0.39	73.8
Sulfadimethoxine	9.02	ND	ND	0.23	25.0
Sulfamethoxazole	8.07	ND	ND	0.24	19.2
Sulfamerazine	6.06	ND	ND	0.12	10.0
Sulfamethazine	328	ND	ND	2.93	24.6
ΣSulfonamides	328	ND	0.64	3.91	86.3
Ofloxacin	75.5	ND	0.13	2.93	50.4
Enrofloxacin	244	ND	ND	6.39	32.1
Norfloxacin	684	ND	0.60	10.1	57.5
Lomefloxacin	18.9	ND	0.16	0.20	53.8
Fleroxacin	28.0	ND	0.54	0.72	58.3
Moxifloxacin	65.0	ND	ND	0.65	2.5
ΣQuinolones	870	ND	2.61	21.0	70.0
Xytetracycline	98.3	ND	ND	4.15	40.0
Tetracycline	40.9	ND	1.01	2.20	63.8
Aureomycin	10.9	ND	4.54	3.29	62.1
ΣTetracyclines	106	ND	6.52	9.65	89.2
Amoxicillin	48.3	ND	ND	8.52	44.6
Cefalexin	56.4	ND	ND	5.84	45.4
Cefotaxime	1.58	ND	ND	0.05	4.2
Cefaclor	21.8	ND	ND	0.65	7.9
Σβ-lactams	99.1	ND	11.6	15.1	70.8
Erythromycin	0.23	ND	ND	0.006	7.5
Azithromycin	6.52	ND	0.22	0.25	62.5
ΣMacrolides	6.52	ND	0.22	0.26	64.6
Lincomycin	0.33	ND	ND	0.03	30.0
Thiamphenicol	6.50	ND	ND	0.18	3.8
ΣAntibiotics	897	ND	28.6	50.0	98.8

^a^ ND, not detected.

**Table 2:** Correlation between livelihood data and total antibiotics in soil in the Pearl River Delta.

Antibiotics	Pig Slaughter Volume	Poultry Slaughter Volume	Aquatic Products Production	Gross Agricultural Output Value	Permanent Resident Population
ΣAntibiotics	0.833 **	0.717 *	0.917 **	0.617	−0.400
Sulfonamides	0.267	0.000	0.233	0.050	−0.233
Quinolones	0.317	0.633	0.733 *	0.233	0.000
Tetracycline	−0.067	−0.583	−0.483	−0.067	−0.033
β-lactams	0.433	−0.083	0.217	0.050	−0.850 **
Macrolides	−0.300	−0.250	−0.517	−0.150	0.217
Lincomycin	−0.333	0.050	0.167	−0.433	0.267
Thiamphenicol	0.424	0.153	0.034	0.797 *	0.153

Note: **, the correlation is significant when the confidence level is 0.01; * When the confidence level (double test) is 0.05, the correlation is significant.

## Data Availability

The data will be made available upon request.

## Supplementary Materials

The following supporting information can be downloaded at: https://www.mdpi.com/article/10.3390/toxics13111004/s1, Text S1: Chemicals and reagents; Text S2: The calibration details and MRM parameter optimization for equipment; Table S1: Analytical parameters of MS for detecting different antibiotics; Table S2: Detection concentration range of antibiotics in farmland soil in different regions of China.
