# Supplementary material for: Soil Antibiotic Pollution and Ecological Risk Assessment in the Pearl River Delta Region, China
Source: Toxics. 2025 Nov 20;13(11):1004. doi: 10.3390/toxics13111004 (PMC12656312; doi:10.3390/toxics13111004)
Supplement: Supplementary file 1 [file toxics-13-01004-s001.zip › toxics-3970463-supplementary.pdf]

Supplementary Material

Soil Antibiotic Pollution and Ecological Risk Assessment in the Pearl River Delta

Region, China

Yong'an Chen [1,2,†], Zhenxian He [1,3,†], Haochuan Wu [4], Xueqin Tao [3], Xiaolong Yu [1,*], Xiaojun Niu [4] and Jianteng Sun [1,*]

1 School of Environmental Science and Engineering, Guangdong University of Petrochemical Technology, Maoming 525000, China; m13790933383_1@163.com (Y.C.); hzxian998@163.com (Z.H.)
2 Chemical Technology and Engineering Faculty, Belarusian State Technological University, 220006 Minsk, Belarus
3 College of Resources and Environment, Zhongkai University of Agriculture and Engineering, Guangzhou 510225, China; xqtao@foxmail.com
4 School of Environment and Energy, South China University of Technology, Guangzhou 510006, China; wuhaochuan2024@163.com (H.W.); xjniu@scut.edu.cn (X.N.)
* Correspondence: yzxendeavour@163.com (X.Y.); sunjianteng@zju.edu.cn (J.S.)
† These authors contributed equally to this work.

Text S1 Chemicals and reagents

Tetracycline antibiotics (oxytetracycline, tetracycline, chlortetracycline; purity ≥95%) were acquired from Shanghai Yuanye Biotechnology Co., Ltd. Macrolide standards (erythromycin, azithromycin; purity ≥98%) were purchased from Shanghai Aladdin Biochemical Technology Co., Ltd. Lincomycin standard (purity ≥98%) was supplied by Shanghai McLean Biochemical Technology Co., Ltd., while thiamphenicol (chloramphenicol antibiotic; purity ≥98%) was procured from Shanghai Yuanye Biotechnology Co., Ltd. KH_2PO_4 (99.9%) and phosphoric acid were provided by Shanghai Aladdin Biochemical Technology Co., Ltd. HLB solid phase extraction column was perchased fromWaters (USA).

Text S2 The calibration details and MRM parameter optimization for equipment

(1) Solution preparation and instrument preparation

Firstly, the standard substance of the antibiotics is prepared into a solution of appropriate concentration (e.g. 1-10 mg/L) for continuous injection into the injection pump. At the same time, the instruments (such as mass spectrometers) were calibrated and gas, voltage, and other parameters are validated for working properly.

(2) Parent ion determination (Q1 Scan)

On the Manual Tuning interface of the mass spectrometer, the injection pump flow rate was adjusted to 5-10 μL/min. Q1 Scan mode were selected and a scanning range that can cover the molecular weight of the tested compound was set. The positive ion scanning mode was adopted. When the total ion chromatogram (TIC) was stabilized, and the parent ion of the target compounds was identified by analyzing the mass spectrum.

(3) Voltage (DP) optimization

After determining the parent ion, Q1 MI (parent ion monitoring) mode was used to input the parent ion, which was optimized by editing Ramp parameters (such as DP voltage). The objective of above process is to achieve the strongest mother ion signal and the lowest noise, determining the optimal DP voltage value.

(4) Determination of sub ions and optimization of collision energy (CE) (Product Ion Scan)

After obtaining the parent ion, the parent ion was input after entering the Product Ion Scan mode, and a scanning range greater than the mass number of the parent ion was set. The optimal value of DP voltage was used, and the initial value of CE (collision energy) can be set to 5 eV. By manually adjusting the CE value, the intensity changes of the parent and daughter ions were monitored. The strength of the daughter ion should be high enough, and the strength of the parent ion should account for about 1/3 to 1/4 of the peak intensity. During this process, all important sub ions was recorded.

(5) MRM parameter optimization and ion pair establishment

In MRM mode, the determined parent and daughter ions was input. CE value and collision chamber exit potential (CXP) value for each sub ion separately were optimized to achieve the best signal-to-noise ratio and response strength through the Ramp function. After optimization is completed, each target ion pair (parent ion/daughter ion) and its corresponding optimal DP, CE, and CXP values were recorded , and the final MRM method was established.

Table S1 Analytical parameters of MS for detecting different antibiotics

Chemicals	Parent ions	Feature fragments	Retention time (min)
Sulfadiazine	251.1482	92.1/156.1	1.57
Sulphadimethoxine	311.04	156/124	3.26
Sulfamethoxazole	254.148	156.0694/92.1/108.0	2.8
Sulfamethazine	279.2	186.2/155/124.2	2.23
Ofloxacin	362	318.1612/261/233.1085	3.48
Enrofloxacin	360.2603	342.2815/316.2	1.9
Norfloxacin	320.2	302.1/231/276.1	3.51
Lomofloxacin	352.3	265.1/308.1	1.78
Fleroxacin	370.2	326.1/269.1	1.53
Oxytetracycline	461.3	425.9/200.9	1.52
Tetracycline	445.3	410.2/154.2	2.61
Chlortetracycline	479	444.1/462.1	2.53
Erythromycin	734.4931	157.9/576.0	3.22
Azithromycin	749.3	591.1/158.0	3.74
Lincomycin	407.5	351/333/126	1.04
Thiamphenicol	354	339/321/293/265/184	8.2

Table S2 Detection concentration range of antibiotics in farmland soil in different regions of China

Sampling Sites	Antibiotics concentration / （μg·kg^{-1}）							Ref.
	ΣSulfonamides	ΣQuinolones	ΣTetracyclines	Σ β-lactams	ΣMacrolides	ΣLincomycin	ΣThiamphenicol	
Guangzhou	—	ND~42.00	—	—	—	—	—	[47]
Pearl River Delta	33.3~321.4	27.8~1537	ND~242.6	—	—	—	—	[39]
Yangtze River Delta	ND~111.0	ND~1410	ND~809.0	—	—	—	—	[42]
Beijing	ND~13.00	ND~649.0	6.1~430.0	—	ND~5.7	—	—	[50]
Chongqing	ND~142.7	ND~485.7	0.8~1195	—	ND~64.80	ND~1.29	ND~5.89	[51]
Zhangjiagang	ND~24.59	ND~458.3	ND~32.65	—	ND~17.66	ND~12.72	ND~114.2	[46]
Pearl River Delta	ND~328	ND~870	ND~106	ND~99.1	ND~6.52	ND~0.33	ND~6.50	Current results
